# Supplementary material for: selSeq: A method for the enrichment of non-polyadenylated RNAs including enhancer and long non-coding RNAs for sequencing
Source: PLoS One. 2023 Nov 28;18(11):e0289442. doi: 10.1371/journal.pone.0289442 (PMC10684010; doi:10.1371/journal.pone.0289442)
Supplement: S1 File — Sequence data is available on SRA under the accession number PRJNA949687. (PDF) [file pone.0289442.s001.pdf]

selSeq: A method for the enrichment of non-polyadenylated RNAs including enhancer and long non-coding RNAs for sequencing

Jason D Limberis[1], Joel Ernst[1], John Metcalfe[1], Alina Nalyvayko[1]

[1]University of California, San Francisco

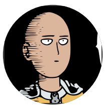

Jason D Limberis
University of California, San Francisco

Protocol Info: Jason D Limberis, Joel Ernst, John Metcalfe, Alina Nalyvayko . selSeq: A method for the enrichment of non-polyadenylated RNAs including enhancer and long non-coding RNAs for sequencing. **protocols.io** https://protocols.io/view/selseq-a-method-for-the-enrichment-of-non-polyaden-crymv7u6

Created: Mar 28, 2023

Last Modified: Jun 12, 2023

PROTOCOL integer ID: 79597

ABSTRACT

Non-polyadenylated RNA includes a large subset of crucial regulators of RNA expression and constitutes a substantial portion of the transcriptome, playing essential roles in gene regulation. For example, enhancer RNAs are long non-coding RNAs that perform enhancer-like functions, are bi-directionally transcribed, and usually lack polyA tails. This paper presents a novel method, *sel*Seq, that selectively removes mRNA and pre-mRNA from samples to enable the selective sequencing of crucial regulatory elements, including non-polyadenylated RNAssuch as long non-coding RNA, enhancer RNA, and non-canonical mRNA.

MATERIALS

Required

- SuperScript® III First-Strand Synthesis System **Thermo Scientific Catalog #18080-051**
- RNase H - 1,250 units **New England Biolabs Catalog #M0297L**
- TURBO DNase 2 U/uL **Fisher Scientific Catalog #AM2239**
- Agencourt RNAClean XP Magnetic Beads **Beckman Coulter Catalog #A63987**
- Ethanol **Contributed by users**

A thermocycler and a qPCR machine
A magnetic rack

Optional

- Luna Universal Probe One-Step RT-qPCR Kit - 200 rxns **New England Biolabs Catalog #E3006S**
- Eukaryotic 18S rRNA Endogenous Control (FAM™/MGB probe, non-primer limited) **Thermo Fisher Catalog #4333760F**
- TaqMan™ GAPDH Control Reagents (human) **Thermo Fisher Catalog #402869**

rRNA depletion oligos

BEFORE START INSTRUCTIONS

Prewarm SuperScript III 10X Buffer to Room temperature

poly-A tailed cDNA synthesis

1 Mix the following in a 0.2ml tube

A	B
Component	Volume (µl)
Total RNA	1
Oligo dTs	1.5
10 mM dNTP mix	1.5
Nuclease-free H2O	10

poly-A tailed cDNA reaction synthesis components

2 Denature sample RNA/primer mixture for 00:05:00 at 65 °C then cool to 4 °C for ≥ 00:02:00 7m

3 Spin tube briefly and add the following and mix by pipetting 55m

A	B
Component	Volume (µl)
10X SuperScript III Buffer	2
25mM MgCl2	4
0.1M DTT	2
Superscript III Reverse Transcriptase	2

poly-A tailed cDNA reaction synthesis components

Incubate 50 °C for 00:50:00 followed by 00:05:00 at 85 °C to deactivate the enzyme, then cool to 4 °C and proceed to the next step

Optional: rRNA depletion

4 Add in the appropriate rRNA depletion oligos for you sample 2m

Incubate 90 °C for 00:02:00 and ramp down to Room temperature at 0.1 °C per second then proceed to the next step

poly-A tailed (and ribosomal) RNA depletion

5 Add 2 µL of RNase H

6 Incubate 37 °C for 00:20:00 followed by 00:05:00 at 65 °C to deactivate the enzyme, then cool it to 4 °C and proceed to the next step 25m

poly-A tailed (and ribosomal) DNA depletion

7 Add in the following components and mix gently by pipetting

A	B
Component	Volume (µl)
10X Turbo DNase Buffer	4
Turbo DNase	4
Nuclease-free H2O	10

DNase treatment components

8 Incubate at 37 °C for 00:30:00 30m

Bead cleanup

9 Add 90 µl (1.8X) of resuspended RNAClean XP Beads to the sample
Mix by pipetting 10x

10 Incubate 00:15:00 at On ice 15m

11 Place on the magnet, allow the beads to aggregate, and remove and discard the supernatant

12 Add 200 µL [M] 80 % (v/v) ethanol and incubate (still on the magnet) for 00:00:30 30s

12.1 Remove the supernatant

12.2 Repeat go to step #12 for a total of 2 washes

13 Air dry for 00:00:30 , don't allow the beads to become cracked 30s

14 Remove the tubes from the magnetic rack
Add 50 µL H20 (optionally add-in 1 µL RNase inhibitor) and resuspend the beads by pipetting ≥10x

15 Incubate 00:05:00 at Room temperature 5m

16 Place on the magnet, aspirate 50 µL of the eluant into a new tube

Optional: One-step RT-qPCR quantification

17

A	B
Component	Volume (µl)
Luna Universal Probe One-Step Reaction Mix (2X)	5
Luna WarmStart RT Enzyme Mix (20X)	0.5
TaqMan GAPDH Control Reagents (human; 20x)	0.5
TaqMan 18S rRNA Control Reagents (eukaryotic; 20x)	0.5
RNA	2
Nuclease-free H2O	1.5

Luna RT-qPCR one-step quantification

18

A	B	C	D	E
Step	Temp (C)	Time (s)	Cycles	Ramp Rate (C/s)
Reverse transcription	55	600	1	2.73
Denaturation	95	60	45	2.73
Denaturation	95	10		2.73
Amplification	60	30		2.11
Capture	60	0		–

Cycle parameters for QuantStudio 3
