# Supplementary material for: selSeq: A method for the enrichment of non-polyadenylated RNAs including enhancer and long non-coding RNAs for sequencing
Source: PLoS One. 2023 Nov 28;18(11):e0289442. doi: 10.1371/journal.pone.0289442 (PMC10684010; doi:10.1371/journal.pone.0289442)
Supplement: Attachment — Submitted filename: Response to Reviewers.docx [file pone.0289442.s002.docx]

PONE-D-23-12460

sel Seq: A method for the enrichment of non-polyadenylated RNAs including enhancer and long non-coding RNAs for sequencing.

PLOS ONE

Dear Dr. Limberis,

Thank you for submitting your manuscript to PLOS ONE. After careful consideration, we feel that it has merit but does not fully meet PLOS ONE’s publication criteria as it currently stands. Therefore, we invite you to submit a revised version of the manuscript that addresses the points raised during the review process.

The comments from two reviewers are at the bottom of the letter. Please address all the points discussed from two reviewers.

Please submit your revised manuscript by Jul 22 2023 11:59PM. If you will need more time than this to complete your revisions, please reply to this message or contact the journal office at [plosone@plos.org](mailto:plosone@plos.org). When you're ready to submit your revision, log on to [https://www.editorialmanager.com/pone/](https://urldefense.com/v3/__https:/www.editorialmanager.com/pone/__;!!LQC6Cpwp!paKRLQ0AmMhUjXXQFm9_n5h0hHpypsrU3VvmlzCDNUzxSsRqHQsrp7J4LKZhm3ewlYXyeZvPX6hM6x_u-jnw_g$) and select the 'Submissions Needing Revision' folder to locate your manuscript file.

Please include the following items when submitting your revised manuscript:

- A rebuttal letter that responds to each point raised by the academic editor and reviewer(s). You should upload this letter as a separate file labeled 'Response to Reviewers'.
- A marked-up copy of your manuscript that highlights changes made to the original version. You should upload this as a separate file labeled 'Revised Manuscript with Track Changes'.
- An unmarked version of your revised paper without tracked changes. You should upload this as a separate file labeled 'Manuscript'.

If you would like to make changes to your financial disclosure, please include your updated statement in your cover letter. Guidelines for resubmitting your figure files are available below the reviewer comments at the end of this letter.

If applicable, we recommend that you deposit your laboratory protocols in protocols.io to enhance the reproducibility of your results. Protocols.io assigns your protocol its own identifier (DOI) so that it can be cited independently in the future. For instructions see: [https://journals.plos.org/plosone/s/submission-guidelines#loc-laboratory-protocols](https://urldefense.com/v3/__https:/journals.plos.org/plosone/s/submission-guidelines*loc-laboratory-protocols__;Iw!!LQC6Cpwp!paKRLQ0AmMhUjXXQFm9_n5h0hHpypsrU3VvmlzCDNUzxSsRqHQsrp7J4LKZhm3ewlYXyeZvPX6hM6x9x4kS5Aw$). Additionally, PLOS ONE offers an option for publishing peer-reviewed Lab Protocol articles, which describe protocols hosted on protocols.io. Read more information on sharing protocols at [https://plos.org/protocols?utm_medium=editorial-email&utm_source=authorletters&utm_campaign=protocols](https://urldefense.com/v3/__https:/plos.org/protocols?utm_medium=editorial-email&utm_source=authorletters&utm_campaign=protocols__;!!LQC6Cpwp!paKRLQ0AmMhUjXXQFm9_n5h0hHpypsrU3VvmlzCDNUzxSsRqHQsrp7J4LKZhm3ewlYXyeZvPX6hM6x_bxunDCg$).

We look forward to receiving your revised manuscript.

Kind regards,

Xiaoyong Sun
Academic Editor
PLOS ONE

Journal requirements:

When submitting your revision, we need you to address these additional requirements.

1. Please ensure that your manuscript meets PLOS ONE's style requirements, including those for file naming. The PLOS ONE style templates can be found at

[https://journals.plos.org/plosone/s/file?id=wjVg/PLOSOne_formatting_sample_main_body.pdf](https://urldefense.com/v3/__https:/journals.plos.org/plosone/s/file?id=wjVg*PLOSOne_formatting_sample_main_body.pdf__;Lw!!LQC6Cpwp!paKRLQ0AmMhUjXXQFm9_n5h0hHpypsrU3VvmlzCDNUzxSsRqHQsrp7J4LKZhm3ewlYXyeZvPX6hM6x9VCEIniA$) and

[https://journals.plos.org/plosone/s/file?id=ba62/PLOSOne_formatting_sample_title_authors_affiliations.pdf](https://urldefense.com/v3/__https:/journals.plos.org/plosone/s/file?id=ba62*PLOSOne_formatting_sample_title_authors_affiliations.pdf__;Lw!!LQC6Cpwp!paKRLQ0AmMhUjXXQFm9_n5h0hHpypsrU3VvmlzCDNUzxSsRqHQsrp7J4LKZhm3ewlYXyeZvPX6hM6x-2MJV4Nw$)

2. We note that you have indicated that data from this study are available upon request. PLOS only allows data to be available upon request if there are legal or ethical restrictions on sharing data publicly. For more information on unacceptable data access restrictions, please see [http://journals.plos.org/plosone/s/data-availability#loc-unacceptable-data-access-restrictions](https://urldefense.com/v3/__http:/journals.plos.org/plosone/s/data-availability*loc-unacceptable-data-access-restrictions__;Iw!!LQC6Cpwp!paKRLQ0AmMhUjXXQFm9_n5h0hHpypsrU3VvmlzCDNUzxSsRqHQsrp7J4LKZhm3ewlYXyeZvPX6hM6x8_xVb1Og$).

In your revised cover letter, please address the following prompts:

a) If there are ethical or legal restrictions on sharing a de-identified data set, please explain them in detail (e.g., data contain potentially sensitive information, data are owned by a third-party organization, etc.) and who has imposed them (e.g., an ethics committee). Please also provide contact information for a data access committee, ethics committee, or other institutional body to which data requests may be sent.

b) If there are no restrictions, please upload the minimal anonymized data set necessary to replicate your study findings as either Supporting Information files or to a stable, public repository and provide us with the relevant URLs, DOIs, or accession numbers. For a list of acceptable repositories, please see [http://journals.plos.org/plosone/s/data-availability#loc-recommended-repositories](https://urldefense.com/v3/__http:/journals.plos.org/plosone/s/data-availability*loc-recommended-repositories__;Iw!!LQC6Cpwp!paKRLQ0AmMhUjXXQFm9_n5h0hHpypsrU3VvmlzCDNUzxSsRqHQsrp7J4LKZhm3ewlYXyeZvPX6hM6x9m1TFGsA$).

We will update your Data Availability statement on your behalf to reflect the information you provide.

[Note: HTML markup is below. Please do not edit.]

Reviewers' comments:

Reviewer's Responses to Questions

**Comments to the Author**


1. Does the manuscript report a protocol which is of utility to the research community and adds value to the published literature?

Reviewer #1: Yes

Reviewer #2: Yes

2. Has the protocol been described in sufficient detail?

To answer this question, please click the link to protocols.io in the Materials and Methods section of the manuscript (if a link has been provided) or consult the step-by-step protocol in the Supporting Information files.

The step-by-step protocol should contain sufficient detail for another researcher to be able to reproduce all experiments and analyses.

Reviewer #1: Yes

Reviewer #2: Yes

3. Does the protocol describe a validated method?

The manuscript must demonstrate that the protocol achieves its intended purpose: either by containing appropriate validation data, or referencing at least one original research article in which the protocol was used to generate data.

Reviewer #1: Yes

Reviewer #2: Yes

4. If the manuscript contains new data, have the authors made this data fully available?

The [PLOS Data policy](https://urldefense.com/v3/__http:/www.plosone.org/static/policies.action*sharing__;Iw!!LQC6Cpwp!paKRLQ0AmMhUjXXQFm9_n5h0hHpypsrU3VvmlzCDNUzxSsRqHQsrp7J4LKZhm3ewlYXyeZvPX6hM6x89fqMjMw$) requires authors to make all data underlying the findings described in their manuscript fully available without restriction, with rare exception (please refer to the Data Availability Statement in the manuscript PDF file). The data should be provided as part of the manuscript or its supporting information, or deposited to a public repository. For example, in addition to summary statistics, the data points behind means, medians and variance measures should be available. If there are restrictions on publicly sharing data—e.g. participant privacy or use of data from a third party—those must be specified.

Reviewer #1: Yes

Reviewer #2: Yes

**5. Is the article presented in an intelligible fashion and written in standard English?**

PLOS ONE does not copyedit accepted manuscripts, so the language in submitted articles must be clear, correct, and unambiguous. Any typographical or grammatical errors should be corrected at revision, so please highlight any specific errors that need correcting in the box below.

Reviewer #1: Yes

Reviewer #2: Yes

We thank the reviewers for their time and insights and thank you for the opportunity to submit a revised protocol. We have carefully considered each comment and made the necessary revisions to strengthen the manuscript. I appreciate your consideration, and we look forward to hearing from you.

6. Review Comments to the Author

Please use the space provided to explain your answers to the questions above. You may also include additional comments for the author, including concerns about dual publication, research ethics, or publication ethics. (Please upload your review as an attachment if it exceeds 20,000 characters)

Reviewer #1: In this paper, the authors found a new method to enrich RNA without polyA tails to facilitate the sequencing of enhancers and non-coding RNAs，which is a meaningful study. However, the following questions remain:
Major Points:

1、There are currently Dynabeads carrying Oligo dTs on the market, which can also remove the RNA of polyA tail, what are the advantages of your method?

selSeq offers several advantages over Dynabeads Oligo dTs. To remove poly-A-tailed RNA using the Dynabeads Oligo dT, the supernatant of the incubation must be kept and used, this would require an additional concentration and cleanup step. If Dynabeads Oligo dTs are used, then the poly-A tailed RNA is removed; however, ribosomal RNA cannot be depleted in the same step as it can in selSeq (step 4 in protocol “Optional: rRNA depletion”). Lastly, using Dynabeads Oligo dTs would increase the cost per sample.

2、In Fig1B, protein RNA accounted for 65.9% after treatment, which is still a high proportion, and I doubt that it can play a huge role in subsequent sequencing work？

We agree. This is most likely comprised of degraded transcripts that lost their poly-A tails or transcripts that lacked poly A tails. Depending on the application of this protocol, it may be helpful in investigating the RNA degradome.

3、Can you use several more genes to verify the efficiency of the your method?

In Figure 1A, we show the results of a qRT-PCR the decrease in polyA-tailed GAPDH housekeeping gene and no decrease in non-polyA tailed 18s ribosomal RNA as described in Step 17 “Optional: One-step RT-qPCR quantification” of the protocol. This is a test to confirm that the protocol worked as expected before sequencing. Since we then sequence, we can compare all genes between the two groups, and determine the reduction in the relative number of reads. For example, if we look at the relative expression for five housekeeping genes (ACTB, GAPDH, RPLP0, TBP, PGK1) between the treatment and no treatment in this example, we find 446 (IQR 388, 597) fold higher *expression* in the No Treatment group. This shows that the method effectively removed these highly expressed, poly-A tailed transcripts.

Reviewer #2: This manuscript describes a novel method for the enrichment of non-polyadenylated RNAs including enhancer and long noncoding RNAs for sequencing.
Two strengths of this manuscript are:
A. A reverse transcriptase quantitative PCR used to see the reduction of polyA housekeeping genes to non-polyA Rrna, but there are hardly any data available to show how relative representation is calculated. The authors describe the reduction in relative expression of GADPH polyA housekeeper genes to non-polyA rRNA, but no description with in-group (treatment) or with intergroup (no treatment&treatment).

We compared the amounts of GADPH and 18s rRNA in different groups by using the Treatment group as a reference. In Figure 1A, we set the relative quantity for the Treatment group as 1, and the error bars represent the variation in the measurements. To calculate this relative quantity, we used a standard method called ΔΔEqCq, which involves determining the fold change between the EqCq values. The EqCq values are obtained by taking the average of the Equivalent Cq values (the PCR cycle number at which the sample's reaction curve intersects the threshold line) for technical replicates of a No Treatment sample and the Treatment reference sample. The summary of these results are in the table below.

| Sample | Target | Delta EqCq Mean | Delta EqCq SD | Delta Delta EqCq | Relative quantity (RQ) | RQ Min | RQ Max |
| --- | --- | --- | --- | --- | --- | --- | --- |
| Treatment | 18s | 10.4 | 0.9 | 0 | 1 | 0.5 | 1.8 |
| Treatment | GAPDH | 24.2 | 1.5 | 0 | 1 | 0.4 | 2.8 |
| No Treatment | 18s | 10.5 | 0.6 | 0.1 | 0.9 | 0.6 | 1.4 |
| No Treatment | GAPDH | 21.6 | 0.1 | -2.6 | 6.1 | 5.7 | 6.6 |

B. Line 56-58 “The proportion of lncRNA, no feature, and miscellaneous RNA have increased substantially, and the protein-coding assignments have decreased, with those remaining likely non-polyadenylated transcripts.” The relative increase times of the proportion of “no feature “is more than that of other types. The author does not explain the potential types of RNA in “no feature” (whether it may be microRNA) and the possible reasons for the large increase times of no feature.

We used the GENOCDE Human GRCh38.p13 genome assembly containing all regions, including reference chromosomes, scaffolds, assembly patches, and haplotypes and the “comprehensive gene annotation”. The reference annotations we used contain many biotypes, including "protein_coding", "lncRNA", "retained_intron", "pseudogenes", "misc_RNA", and "miRNA". Any sequence that aligns to the reference but contains no information in the annotation file is classified as “no feature”. Since the annotations of non-coding RNAs are not exhaustive, as methods to detect and classify them are few, we think that most of the increase in this group is due to unannotated non-coding RNAs.

A smattering of suggested corrections:
1. Figure 1B, the misspelling “no feaure”.
2. In the Supporting Information, the misspelling “Optional: Ane-step RT-qPCR quantification” in the last step.

Thanks, we have corrected these.

7. PLOS authors have the option to publish the peer review history of their article ([what does this mean?](https://urldefense.com/v3/__https:/journals.plos.org/plosone/s/editorial-and-peer-review-process*loc-peer-review-history__;Iw!!LQC6Cpwp!paKRLQ0AmMhUjXXQFm9_n5h0hHpypsrU3VvmlzCDNUzxSsRqHQsrp7J4LKZhm3ewlYXyeZvPX6hM6x8zA8dSiQ$)). If published, this will include your full peer review and any attached files.

If you choose “no”, your identity will remain anonymous but your review may still be made public.

**Do you want your identity to be public for this peer review?** For information about this choice, including consent withdrawal, please see our [Privacy Policy](https://urldefense.com/v3/__https:/www.plos.org/privacy-policy__;!!LQC6Cpwp!paKRLQ0AmMhUjXXQFm9_n5h0hHpypsrU3VvmlzCDNUzxSsRqHQsrp7J4LKZhm3ewlYXyeZvPX6hM6x8RyfkhvA$).

Reviewer #1: No

Reviewer #2: **Yes:** Xinhua Ding

[NOTE: If reviewer comments were submitted as an attachment file, they will be attached to this email and accessible via the submission site. Please log into your account, locate the manuscript record, and check for the action link "View Attachments". If this link does not appear, there are no attachment files.]

While revising your submission, please upload your figure files to the Preflight Analysis and Conversion Engine (PACE) digital diagnostic tool, [https://pacev2.apexcovantage.com/](https://urldefense.com/v3/__https:/pacev2.apexcovantage.com/__;!!LQC6Cpwp!paKRLQ0AmMhUjXXQFm9_n5h0hHpypsrU3VvmlzCDNUzxSsRqHQsrp7J4LKZhm3ewlYXyeZvPX6hM6x_4wwTnpw$). PACE helps ensure that figures meet PLOS requirements. To use PACE, you must first register as a user. Registration is free. Then, login and navigate to the UPLOAD tab, where you will find detailed instructions on how to use the tool. If you encounter any issues or have any questions when using PACE, please email PLOS at [figures@plos.org](mailto:figures@plos.org). Please note that Supporting Information files do not need this step.
